# Exploring Associations Among Loneliness, Depression, and Cardiovascular Risk Factors in a Free Medical Clinic: A Cross-Sectional Pilot Study

**DOI:** 10.7759/cureus.112855

**Published:** 2026-07-17

**Authors:** Bailey Cook, Christian Zanetti-Weeks, Mary Price, Laken E Fulmer, Lisa Carroll

**Affiliations:** 1 Clinical Sciences, Edward Via College of Osteopathic Medicine, Spartanburg, USA; 2 Medicine, Edward Via College of Osteopathic Medicine, Spartanburg, USA; 3 Family Medicine, Edward Via College of Osteopathic Medicine, Spartanburg, USA

**Keywords:** atherosclerotic cardiovascular disease, cardiovascular disease risk, depression, free medical clinic, hypertension, loneliness, total cholesterol

## Abstract

Introduction

Depression and loneliness have been linked to cardiovascular disease (CVD), but few studies explore their combined impact. Loneliness has been identified as an independent CVD risk factor, while depression has been tied to elevated CVD risk. This study examined relationships among University of California-Los Angeles (UCLA) Loneliness Scale scores, Patient Health Questionnaire-9 (PHQ-9) depression scores, and CVD risk factors in patients at a free medical clinic in South Carolina.

Methods

Thirty-one English-speaking adults aged 20-79 with a recent lipid panel completed the PHQ-9 and UCLA Loneliness Scale. Scores were calculated per standard guidelines, and lifetime Atherosclerotic Cardiovascular Disease (ASCVD) risks were computed using the American College of Cardiology (ACC) ASCVD calculator. Pearson correlations, multiple regressions, and t-tests were performed to explore relationships. Subgroup sizes varied for analysis depending on the availability of UCLA Loneliness data, permissible lipid profiles, and unreliable psychosocial responses.

Results

Multiple regression showed PHQ-9 and UCLA Loneliness scores significantly predicted total cholesterol (TCHOL) (R²=0.27, p=0.024). This association was stronger among non-statin users (n=14; R²=0.54, p=0.014), where PHQ-9 was inversely associated with TCHOL (b=-11.9, p=0.0044) and UCLA Loneliness score was positively associated with TCHOL (b=5.69, p=0.0056), although small sample instability substantially confines this analysis. A similar pattern was observed for high-density lipoprotein (HDL) among non-statin users (R²=0.44, p=0.04). PHQ-9 and UCLA Loneliness scores were strongly correlated (R=0.70, 95% CI: (0.43, 0.85), p<0.0001, n=27). These findings should be interpreted with caution, as relationships may have been inflated by multicollinearity, especially in non-statin group analyses. Current/former smokers had significantly higher PHQ-9 (p=0.009) and UCLA Loneliness scores (p=0.04) compared to never-smokers. Correlations among PHQ-9, UCLA scores, and ASCVD lifetime risk among non-statin users were weak and nonsignificant (PHQ-9: R=0.165, p=0.574; UCLA: R=0.280, p=0.404).

Conclusion

While no significant associations were observed between psychosocial scores and ASCVD lifetime risk, significant associations among depression, loneliness, and lipid values, particularly among non-statin users, along with associations between psychosocial scores and smoking status, suggest a potential link between mental health and cardiovascular risk factors. Larger studies are warranted to confirm and quantify these relationships.

## Introduction

Heart disease is the leading cause of death in the United States [1]. Many risk factors have been identified which contribute to the onset of heart disease, including age greater than 60, diabetes, hyperlipidemia (HLD), and hypertension (HTN) [2,3]; however, mental health factors are often overlooked. Previous studies have suggested associations with depression [4] and loneliness [5,6] and an increased risk of the development of coronary artery disease (CAD). Furthermore, studies have found loneliness and depression to be correlated [7].

It has been well documented that dyslipidemia, including but not limited to elevated total cholesterol (TCHOL), elevated low-density lipoprotein (LDL), and decreased high-density lipoprotein (HDL), is a major risk factor for CAD [2,3,8]. Further studies have found positive correlations between hyperlipidemia and major depression [9] and between metabolic syndrome and loneliness [10]. Interestingly, some studies have shown decreased TCHOL levels to be related to a higher risk for suicide [11]. Regardless of the effect, there is an evident relationship between cholesterol levels and mental health.

Psychological disorders such as depression, post-traumatic stress disorder (PTSD), anxiety, and stress have been shown to have a neurobiological effect on the body, particularly in women. A study published in 2016 found that all of these emotional states had a significant impact on women’s cardiovascular health, citing the hypothalamic-pituitary axis and norepinephrine system as possibly contributing to the development of microvascular disease and endothelial dysfunction leading to poor cardiovascular health outcomes in women. Although depression was mentioned among the disorders in this particular study, no unique biophysiological reason for the effect on cardiovascular health was mentioned as the researchers stated further studies were warranted [12]. Another study published in 2023 looked at the biophysiological effect of PTSD and trauma on women’s cardiovascular health in greater depth. In this particular study, researchers cited immune system dysregulation, increased inflammation, oxidative stress and mitochondrial dysfunction, renin-angiotensin system dysregulation, and accelerated biological aging as possible pathways leading to the development of CVD from acute stress and PTSD [13]. Previous articles also found associations between PTSD and increased CVD risk in males, particularly veterans [14]. These studies did not look at depression or loneliness specifically, illustrating that further investigation is needed in these areas. However, these results suggest that mental states can affect biophysiological processes which can lead to the development of CVD.

Despite evidence of the relationship between loneliness and depression with heart disease, there have been few published cross-sectional studies exploring the relationship among loneliness, depression, and heart disease risk factors in both males and females. One study published in 2021 found that genetic markers for both major depressive disorder and loneliness were associated with the incidence of CAD in females via retrospective chart review [15]. Another study published in 2024 reviewed recent evidence regarding the impacts of several different mental health disorders including depression, social isolation and loneliness, anxiety, PTSD, stress, and anger on women’s cardiovascular health, emphasizing the importance of mental health screening tools as a preventative measure for ischemic heart disease (IHD) [16]. Similarly, a meta-analysis published in 2019 reviewed the effect various mental health disorders had on the incidence of CAD in men and women. Interestingly, the researchers found no significant difference in cardiovascular outcomes between the two groups, but noted that depression, anxiety, and distress affected cardiovascular outcomes in both men and women, while anger and hostility affected outcomes in men only. Suboptimal social support did not seem to significantly affect the rates of IHD in either group. However loneliness, being distinct from lack of social support, was not a specific variable that was studied [17]. The purpose of this cross-sectional pilot study is to explore the relationship between and generate hypotheses on mental health and heart disease utilizing validated surveys to screen for depression, loneliness, and heart disease risk and associated cardiovascular factors (such as lipid profiles and smoking status) in order to determine whether correlations exist between these variables among male and female patients at a free medical clinic in South Carolina, in the hopes that larger studies may be performed thereafter. This article was previously presented as a poster at the 2025 American College of Osteopathic Internists (ACOI) Annual Conference on October 10, 2025, and to the 2026 ACOFP Osteopathic Clinical Research Committee on January 13, 2026.

## Materials and methods

A cross-sectional three-variable correlational study was conducted from May through June 2025, utilizing a survey which included demographics and past medical and mental health history questions, as well as the Patient Health Questionnaire-9 (PHQ-9) [18], and the University of California-Los Angeles (UCLA) Loneliness Scale [19], both validated surveys for depression and loneliness screening and grading, respectively (Appendix 1). The American College of Cardiology (ACC) Atherosclerotic Cardiovascular Disease (ASCVD) online risk calculator was used to calculate lifetime risks of CVD based on participant age, sex, race, and risk factors including history of type 2 diabetes, hypertension, and smoking, recent TCHOL, LDL and HDL levels, current systolic and diastolic blood pressure (BP) readings, and use of antihypertensives, statins, and aspirin (Appendix 2) [1,2].

Thirty-one participants joined this study among patients at a free medical clinic who met the inclusion criteria of being between the ages of 20 and 79, English-speaking, and had a lipid panel drawn within the last year. Qualifying patients were asked if they would like to participate in a brief survey related to mental health and heart disease risk, with the incentive of a free pulse oximeter for their participation (Appendix 3). Formal tracking of the number of patients approached or who declined participation was not conducted, representing a limitation of the recruitment process. Upon receiving written consent, results from the participants’ most recent lipid panel were recorded as well as their BP reading from that day. Each participant was asked to fill out the survey described above. Investigators then utilized the ACC ASCVD risk calculator to estimate lifetime risk of heart disease. PHQ-9 depression scores were calculated by summing responses across all nine items, yielding scores ranging from 0 to 27, per standard scoring guidelines. UCLA Loneliness Scale scores were calculated per the instrument's published scoring protocol. Higher scores on both instruments indicate greater severity of depression and loneliness, respectively. De-identified participant responses were recorded into a spreadsheet to which only the investigators had access.

Of 31 enrolled participants, one (participant #1) was excluded from all psychosocial analyses due to an invariant response pattern, defined as identical responses selected across all items of both the PHQ-9 and UCLA Loneliness Scale, which is indicative of non-differentiated or inattentive responding and renders the resulting scores uninterpretable. Three additional participants (#25, #28, #29) provided incomplete UCLA Loneliness Scale responses and were excluded from any analysis requiring UCLA scores. One participant (#31) had an outdated lipid panel and was excluded from lipid-related analyses. No imputation of missing values was performed; participants with missing data on any variable required for a given analysis were excluded from that analysis only, with analytic sample sizes varying accordingly.

These exclusions yielded the following analytic samples: lipid regression analyses used n=26 (excluding #1, #25, #28, #29, and #31); analyses involving UCLA Loneliness Scale scores and smoking history used n=27 (excluding #1, #25, #28, and #29 only, as #31's lipid panel was not required for this analysis); and ASCVD lifetime risk analyses were further restricted to non-statin users who met the ACC ASCVD calculator's eligibility criteria, as the calculator is not intended for use in patients on statin therapy or those with lipid values outside the calculator's accepted parameters. Specific participant exclusions for each analysis are noted in the corresponding tables. The statistical power and generalizability of findings are substantially limited by the small sample size and subsequent subgroup analyses.

To evaluate associations among study variables, t-tests were applied using Microsoft Excel (Microsoft Corporation, Redmond, Washington, USA) to compare cardiovascular and psychosocial outcomes by smoking history. Welch’s t-tests were specifically employed due to unequal variance assumptions when comparing these indicators across smoking history groups. Pearson correlations were calculated and a scatterplot was generated using Excel comparing PHQ-9 and UCLA Loneliness Scale scores. Multiple linear regression models were specified with lipid markers (TCHOL, HDL, LDL) as dependent variables and PHQ-9 and UCLA Loneliness Scale scores as independent variables, entered simultaneously in a single block. Models were run for the aggregate sample and stratified by statin use. Sensitivity analyses were subsequently conducted using simple linear regressions with each psychosocial predictor entered as the sole independent variable for each lipid outcome and subgroup. Formal assumption testing for regression models was not conducted, representing a methodological limitation consistent with the exploratory pilot design. All statistical analyses were performed using Microsoft Excel, including the Data Analysis ToolPak for regression analyses, the CORREL function for Pearson correlations, and Welch's t-tests for group comparisons. Variance Inflation Factors (VIF) diagnostics were calculated directly from inter-predictor correlations using the formula VIF = 1/(1-R²), where R represents the Pearson correlation between PHQ-9 and UCLA Loneliness Scale scores within each analytic subgroup.

Each participant was offered a flyer including a list of both national and local mental health resources (Appendix 4). Participants with at least moderate depression and/or loneliness who reported not currently being treated for depression with therapy and/or medication were called at the conclusion of the study to ensure they received a flyer and offer further resources, including counseling which is offered at the free clinic. All participants were also given the opportunity to give their written consent to share their results with their provider to be reviewed at their next visit. The medical director of the free clinic was also made aware of any significant findings.

Post hoc power analyses were conducted using WebPower (University of Notre Dame, Notre Dame, Indiana, USA) [20] and the Cleveland Clinic Sample Size Calculator [21] to determine adequate sample sizes for each analysis type (α=0.05, power=0.80, two-sided). Required sample sizes varied substantially by analysis: approximately 68 participants for the primary multiple linear regression model assuming a medium effect size (f²=0.15), 41-63 total participants for Welch's t-tests comparing smoking groups (d=1.04 and d=0.80 for PHQ-9 and UCLA Loneliness Scale, respectively), and 13-285 participants for Pearson correlations (R=0.70 to R=0.165). The enrolled sample of 31 participants (with less than that excluded for analysis) met power thresholds only for the PHQ-9/UCLA Loneliness Scale correlation and was substantially underpowered for the ASCVD correlation analyses, given the weak observed correlations in this domain. Subsequently, results should be interpreted with caution as the analyses were underpowered overall. This study was therefore designed as a pilot to generate hypotheses and preliminary effect size estimates to inform adequately powered follow-up studies, with recruitment constrained by available clinic days and an English-speaking-only inclusion criterion. This project was approved by Edward Via College of Osteopathic Medicine’s Institutional Review Board, IRB number 2302212-2, dated: April 30, 2025. Funding was obtained from Edward Via College of Osteopathic Medicine (VCOM)-Carolinas Campus through the Biomedical Department research fund. Informed written consent was obtained by the investigators from all participants prior to survey completion.

## Results

A total of 31 participants were enrolled in this study from a free clinic setting (17 women and 14 men). Race data was self-reported as Asian (n=2), Black (n=10), Hispanic (n=3), and White (n=16). Most individuals fell within the 40-69 year age range. Smoking history was reported using the following categories: current smoker (n=11), former smoker (n=9), and never smoker (n=11). Participants also self-reported cardiovascular treatment history, including aspirin use (n=12), statin therapy (n=13), or treatment for hypertension (HTN) (n=17). One participant was consistently excluded from the analyses below due to unreliable psychosocial survey response patterns and missing laboratory data required for analysis (participant #1).

Welch's t-tests were used to compare PHQ-9 and UCLA Loneliness scores across smoking history groups. Current/former smokers (n=17) had significantly higher PHQ-9 scores than never-smokers (n=10) (M=10.6, SD=8.12 vs. M=4, SD=3.77; mean difference=6.59, 95% CI: (1.83, 11.3), t(24)=2.86, p=0.009, d=1.04). Similarly, current/former smokers had significantly higher UCLA Loneliness scores than never-smokers (M=41.2, SD=17.6 vs. M=30.1, SD=8.72; mean difference=11.1, 95% CI: (0.61, 21.5), t(25)=2.18, p=0.04, d=0.80).

Exploratory pairwise comparisons among current, former, and never-smokers (Table 1) suggested that these effects were driven primarily by current smokers. Current smokers had significantly higher PHQ-9 scores than both former smokers (mean difference=9.4, 95% CI: (2.5, 16.3), t(11)=2.99, p=0.012, d=2.78) and never-smokers (mean difference=11, 95% CI: (4.11, 17.9), t(11)=3.51, p=0.005, d=1.64), while former and never-smokers did not differ significantly (p=0.350). A similar pattern was observed for UCLA Loneliness Scale scores, with current smokers showing significantly higher loneliness scores than never-smokers (mean difference=18.7, 95% CI: (5.16, 32.2), t(12)=3.01, p=0.011, d=1.40), while the current-versus-former and former-versus-never comparisons did not reach significance (p=0.054 and p=0.684, respectively) (Table 1). No significant differences in UCLA Loneliness and PHQ-9 scores were found between self-identified former smokers and never-smokers. These comparisons based upon smoking status were evaluated after excluding participants who did not complete the UCLA Loneliness scale in its entirety (patients #25, 28, and 29).

**Table 1:** Welch’s t-Test Results for PHQ-9 Depression and UCLA Loneliness Scores by Smoking Status *Indicates significant relationship (p<0.05). PHQ-9: Patient Health Questionnaire-9, UCLA: University of California-Los Angeles.

Psychosocial Measure	Comparison	n_1_	n_2_	Mean_1_	Mean_2_	SD_1_	SD_2_	Mean Difference	95% CI	t	df	p value	Cohen’s d
PHQ-9	Current vs. Former	9	8	15	5.63	8.7	3.4	9.4	(2.5, 16.3)	2.99	11	0.012*	2.78
	Current vs. Never	9	10	15	4	8.7	3.77	11	(4.11, 17.9)	3.51	11	0.005*	1.64
	Former vs. Never	8	10	5.63	4	3.38	3.77	1.63	(-1.95, 5.41)	0.963	16	0.350	0.454
	Current/Former vs. Never	17	10	10.6	4	8.12	3.77	6.59	(1.83, 11.3)	2.86	24	0.009*	1.04
UCLA	Current vs. Former	9	8	48.8	32.6	16.7	15.2	16.2	(-0.34, 32.6)	2.09	15	0.054	1.01
	Current vs. Never	9	10	48.8	30.1	16.7	8.72	18.7	(5.16, 32.2)	3.01	12	0.011*	1.40
	Former vs. Never	8	10	32.6	30.1	15.2	8.72	2.53	(-10.8, 15.8)	0.417	11	0.684	0.202
	Current/Former vs. Never	17	10	41.2	30.1	17.6	8.72	11.1	(0.61, 21.5)	2.18	25	0.04*	0.80

A multiple linear regression model was used to evaluate the relationship between lipid values and psychosocial screening scores (PHQ-9 and the UCLA Loneliness Scale). Participants with unreliable psychosocial screening answers, outdated lipid panel values, and incomplete UCLA loneliness scale responses were excluded from the following analyses. When examining TCHOL’s relationship with PHQ-9 and UCLA loneliness scale, the overall model was statistically significant and explained a moderate proportion of variance (R^2^=0.27, adjusted R^2^=0.21, F(2,23)=4.42, p=0.024, n=26), although these findings should be interpreted cautiously given the absence of correction for multiple comparisons. Both predictors contributed significantly to the model with TCHOL. PHQ-9 scores were inversely associated with TCHOL (b=-5.52, 95% CI: (-9.50, -1.54), p=0.0087], indicating that for each one-point increase in PHQ-9 score, TCHOL decreased by approximately 5.5 mg/dL (95% CI: (1.5, 9.5) mg/dL). UCLA Loneliness scores were positively associated with TCHOL (b=2.34, 95% CI: (0.45, 4.23), p = 0.017), corresponding to an increase of approximately 2.3 mg/dL in TCHOL per one-point increase in UCLA Loneliness score (95% CI: (0.5, 4.2) mg/dL) (Table 2).

**Table 2:** Summary of Multiple Regression Models Predicting Lipid Values from PHQ-9 and UCLA Loneliness Scores Excluded participants #1 (concerns over psychosocial response reliability), #25, 28, 29 (missing UCLA data), and #31 (outdated lipid panel). *Indicates significant relationship (p<0.05). PHQ-9: Patient Health Questionnaire-9, UCLA: University of California-Los Angeles, VIF: Variance Inflation Factors, TCHOL: total cholesterol, LDL: low-density lipoprotein, HDL: high-density lipoprotein.

Lipid Marker	Group	n	R^2^	Adjusted R^2^	F	df1	df2	p-value (model)	VIF
TCHOL	Aggregate	26	0.27	0.21	4.42	2	23	0.024*	1.94
	Statin Users Only	12	0.35	0.21	2.44	2	9	0.143	1.25
	Non-Statin Users Only	14	0.54	0.46	6.51	2	11	0.014*	5.16
LDL	Aggregate	26	0.008	-0.078	0.098	2	23	0.91	1.94
	Statin Users Only	12	0.08	-0.131	0.365	2	9	0.704	1.25
	Non-Statin Users Only	14	0.07	-0.10	0.387	2	11	0.69	5.16
HDL	Aggregate	26	0.15	0.08	2.07	2	23	0.15	1.94
	Statin Users Only	12	0.28	0.12	1.75	2	9	0.23	1.25
	Non-Statin Users Only	14	0.44	0.34	4.39	2	11	0.04*	5.16

This relationship was strengthened when stratified by statin use in the non-statin users subgroup, but not in the statin users subgroup. Among non-statin users (n=14), the model explained a substantially larger proportion of variance in TCHOL (R²=0.54, adjusted R²=0.46, F(2,11)=6.51, p=0.014), with both PHQ-9 (b=-11.9, 95% CI: (-19.3, -4.57), p=0.0044) and UCLA Loneliness scores (b=5.69, 95% CI: (2.04, 9.33), p=0.0056) showing stronger associations with TCHOL than in the aggregate model, though should be carefully interpreted due to the small subgroup size. In contrast, the model did not reach significance among statin users (n=12; R²=0.35, adjusted R²=0.21, F(2,9)=2.44, p=0.143) (Tables 2, 3). Omnibus models for LDL were nonsignificant across the aggregate sample and both statin-use strata (all p>0.6) (Table 2). For HDL, the aggregate and statin-user models were also nonsignificant; however, among non-statin users (n=14), the overall model reached statistical significance (R²=0.44, adjusted R²=0.34, F(2,11)=4.39, p=0.04). In this subgroup, PHQ-9 was inversely associated with HDL (b=-9.53, 95% CI: (-16.6, -2.44), p=0.013), and UCLA Loneliness scores were positively associated with HDL (b=4.36, 95% CI: (0.84, 7.87), p=0.02) (Tables 2, 3).

**Table 3:** Multiple Regression Coefficients for PHQ-9 and UCLA Loneliness Scores as Predictors of Lipid Values Excluded participants #1 (concerns over psychosocial response reliability), #25, 28, 29 (missing UCLA data), and #31 (outdated lipid panel). *Indicates significant relationship (p<0.05). PHQ-9: Patient Health Questionnaire-9, UCLA: University of California-Los Angeles, TCHOL: total cholesterol, LDL: low-density lipoprotein, HDL: high-density lipoprotein.

Lipid Marker	Group	n	PHQ-9 b	PHQ-9 95% CI	PHQ-9 p-value	UCLA b	UCLA 95% CI	UCLA p-value
TCHOL	Aggregate	26	-5.52	(-9.50, -1.54)	0.0087*	2.34	(0.45, 4.23)	0.017*
	Statin Users Only	12	-3.85	(-8.73, 1.04)	0.108	1.92	(-0.29, 4.15)	0.081
	Non-Statin Users Only	14	-11.9	(-19.3, -4.57)	0.0044*	5.69	(2.04, 9.33)	0.0056*
LDL	Aggregate	26	-0.18	(-1.04, 0.67)	0.663	0.06	(-0.34, 0.47)	0.196
	Statin Users Only	12	-0.43	(-1.62, 0.75)	0.431	0.041	(-0.50, 0.58)	0.869
	Non-Statin Users Only	14	0.11	(-1.45, 1.68)	0.88	0.085	(-0.69, 0.86)	0.814
HDL	Aggregate	26	-3.46	(-7.21, 0.30)	0.07	1.59	(-0.20, 3.37)	0.08
	Statin Users Only	12	-1.20	(-5.27, 2.88)	0.52	1.52	(-0.33, 3.38)	0.10
	Non-Statin Users Only	14	-9.53	(-16.6, -2.44)	0.013*	4.36	(0.84, 7.87)	0.02*

To assess multicollinearity between PHQ-9 and UCLA Loneliness Scale scores, Variance Inflation Factors (VIF) were calculated for each multiple regression model (Table 2). VIF values were 1.94 in the aggregate sample (n=26), 1.25 in the statin-user subgroup (n=12), and 5.16 in the non-statin subgroup (n=14), reflecting the inter-predictor correlations of R=0.70, R=0.45, and R=0.90, respectively. Sensitivity analyses were conducted using simple linear regressions with PHQ-9 and UCLA Loneliness scores entered as sole predictors of each lipid outcome. Across all outcomes (TCHOL, HDL, LDL) and all subgroups, no simple regression model reached statistical significance (Table 4).

**Table 4:** Simple Regression Coefficients for PHQ-9 and UCLA Loneliness Scores as Predictors of Lipid Values Excluded participants #1 (concerns over psychosocial response reliability), #25, 28, 29 (missing UCLA data), and #31 (outdated lipid panel). PHQ-9: Patient Health Questionnaire-9, UCLA: University of California-Los Angeles, TCHOL: total cholesterol, LDL: low-density lipoprotein, HDL: high-density lipoprotein.

Lipid Marker	Group	n	PHQ-9 R^2^	PHQ-9 b	95% CI	PHQ-9 p-value	UCLA R^2^	UCLA b	95% CI	UCLA p-value
TCHOL	Aggregate	26	0.072	-2.09	(-5.25, 1.08)	0.187	0.02	0.52	(-1.03, 2.06)	0.496
	Statin Users Only	12	0.074	-1.96	(-6.84, 2.92)	0.392	0.123	1.145	(-1.01, 3.31)	0.265
	Non-Statin Users Only	14	0.052	-1.63	(-6.05, 2.78)	0.435	0.011	0.374	(-1.86, 2.61)	0.722
LDL	Aggregate	26	0.004	-0.092	(-0.69, 0.51)	0.753	2.02 x 10^-6^	0.001	(-0.28, 0.29)	0.995
	Statin Users Only	12	0.072	-0.392	(-1.38, 0.60)	0.399	0.005	-0.05	(-0.51, 0.42)	0.827
	Non-Statin Users Only	14	0.061	0.265	(-0.39, 0.92)	0.395	0.064	0.135	(-0.19, 0.46)	0.384
HDL	Aggregate	26	0.028	-1.13	(-3.95, 1.69)	0.415	0.019	0.443	(-0.90, 1.79)	0.503
	Statin Users Only	12	0.003	0.302	(-3.71, 4.31)	0.870	0.245	1.283	(-0.30, 2.87)	0.102
	Non-Statin Users Only	14	0.068	-1.65	(-5.48, 2.18)	0.366	0.001	0.109	(-1.86, 2.08)	0.906

Among non-statin users, no significant correlations were observed between psychosocial scores and ASCVD lifetime risk. PHQ-9 scores showed a weak, nonsignificant positive correlation with ASCVD lifetime risk (R=0.165, 95% CI: (-0.40, 0.64), p=0.574, n=14). Similarly, UCLA Loneliness scores showed a weak, nonsignificant positive correlation with ASCVD lifetime risk (R=0.280, 95% CI: (-0.38, 0.75), p=0.404, n=11) (Table 5). Relationships between psychosocial scores were weak and nonsignificant.

**Table 5:** Associations Between PHQ-9, UCLA Loneliness Scores, and ASCVD Lifetime Risk Among Non-Statin Users ^Note that ASCVD risk calculators are NOT meant to be used for patients with ASCVD or those who are on LDL-reducing treatment. The results in this table exclude participants 1, 2, 4, 5, 8, 10, 11, 12, 14, 15, 17, 18, 23, 24, 26, 30, and 31 (could not calculate lifetime risk due to patient factors (e.g., statin use, age, and lipid values outside of the calculator’s parameters). #Excludes participants listed above along with patients 25, 28, and 29 due to incomplete UCLA loneliness scale responses. PHQ-9: Patient Health Questionnaire-9, UCLA: University of California-Los Angeles, CVD: cardiovascular disease, ASCVD: Atherosclerotic Cardiovascular Disease, LDL: low-density lipoprotein.

CVD Risk Measurement	Psychosocial Measure	n	R	95% CI	p-Value
ASCVD Lifetime Risk (Non-Statin Users Only)	^PHQ-9	14	R=0.165	(-0.40, 0.64)	0.574
	^^#^UCLA	11	R=0.280	(-0.38, 0.75)	0.404

**Figure 1:**
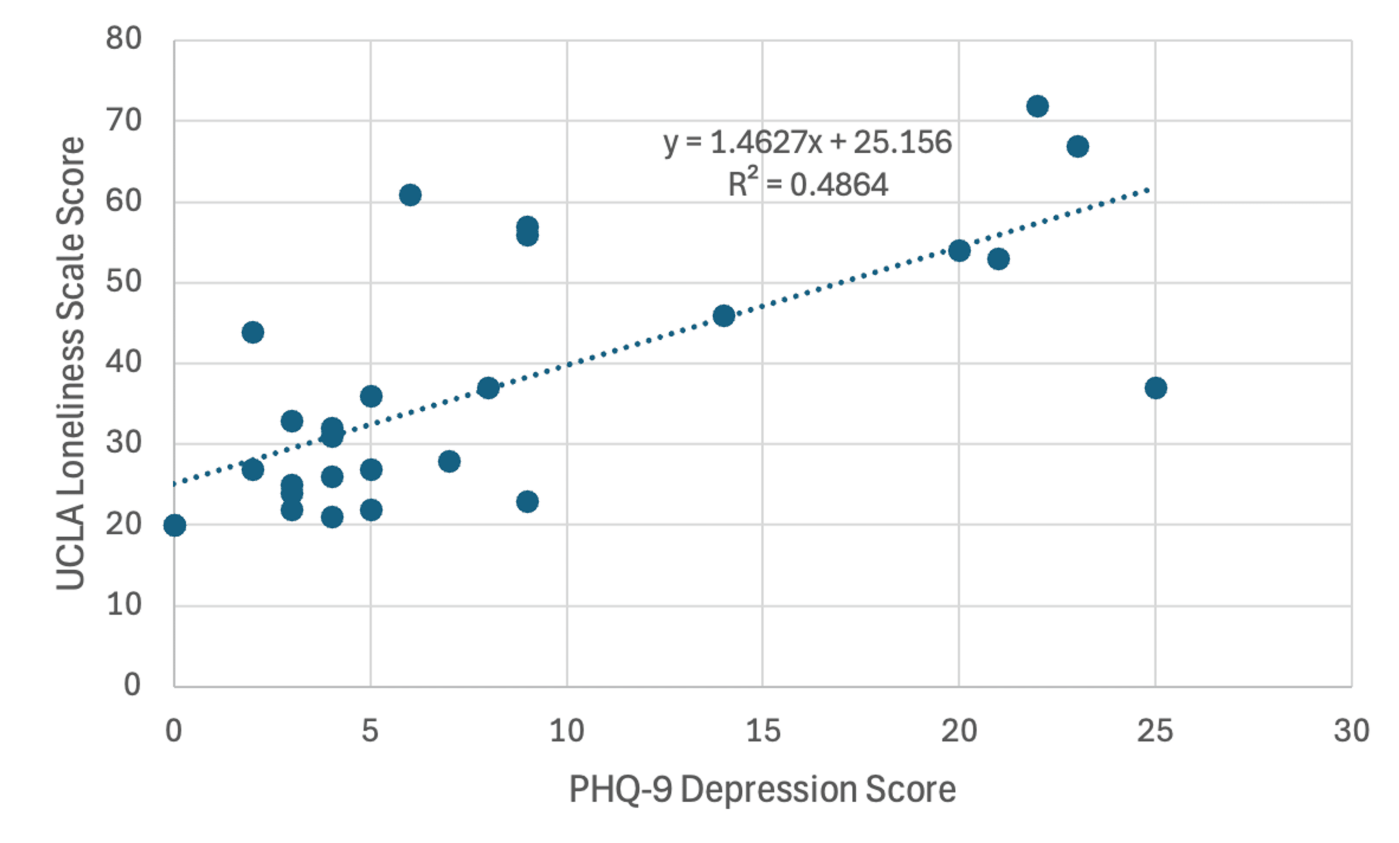
Relationship Between Depressive Symptoms and Loneliness in the Study Population Higher PHQ-9 scores were significantly associated with higher UCLA Loneliness Scale scores (R=0.70, 95% CI: (0.43, 0.85), p<0.0001, n=27). PHQ-9: Patient Health Questionnaire-9, UCLA: University of California-Los Angeles.

A positive correlation between PHQ-9 depression scores and UCLA Loneliness Scale scores was statistically significant (R=0.70, 95% CI: (0.43, 0.85), p<0.0001, n=27), after excluding patients #25, 28, and 29 with incomplete UCLA Loneliness Scale results (Figure 1).

## Discussion

Pearson correlation analysis revealed no significant relationships among depression, loneliness, and lifetime ASCVD risk scores, likely due to small sample sizes and subgroups. However, a significant positive correlation was found between loneliness and depression, consistent with previous studies [6]. Multiple linear regression revealed significant associations between psychosocial scores and total cholesterol (TCHOL): PHQ-9 was inversely associated with TCHOL, while UCLA Loneliness scores were positively associated with TCHOL. Stratification by statin use did not entirely weaken these associations; rather, the relationship between psychosocial scores and TCHOL was substantially stronger among non-statin users than in the aggregate sample. This pattern suggests that statin therapy itself may attenuate the obscure relationship between psychosocial factors and lipid values, although this cannot be confirmed given the small subgroup sizes. Furthermore, stratification by statin use was prioritized due to their direct effect on cholesterol values and subsequent analysis between the psychosocial measures and participants' lipid profiles. Similarly, in non-statin users, both PHQ-9 scores and UCLA Loneliness scores were significantly associated with HDL, with a pattern consistent with that observed for TCHOL. Given that multiple comparisons were conducted without correction, the probability of at least one false positive finding is elevated, and these results should be considered hypothesis-generating rather than confirmatory.

Although no significant correlations were found among depression, loneliness, and lifetime ASCVD risk scores, the significant correlation among depression, loneliness, and TCHOL levels suggests a connection between these psychosocial factors and CVD risk. Increased TCHOL levels are a major risk factor for CAD [2,7], and the findings in this study suggest that higher loneliness scores correlate with higher TCHOL levels, which is consistent with previous findings [9]. The results from this study suggest a negative correlation between depression risk scores and TCHOL, in contrast to prior research [10]. These preliminary findings, particularly the strengthened associations observed in the non-statin subgroup, provide effect size estimates that could inform power calculations for future, larger studies specifically designed to examine these relationships within statin-use strata. Stratification should also occur across other variables such as age, sex, race, smoking status, body mass index (BMI), and diagnoses of hypertension and diabetes.

Additionally, higher depression risk and loneliness scores observed among participants with a smoking history are consistent with literature indicating that smoking is associated with greater psychosocial distress, including depression [22] and loneliness [23]. Notably, mean psychosocial measures were fairly similar between the former smoker and non-smokers, suggesting that more optimal psychosocial measures could be associated with smoking abstinence. However, the literature suggests that former smokers may still experience greater risk of cardiovascular disease than never-smokers [24], despite their similar psychosocial scores.

Limitations

This pilot study was conducted at a free medical clinic which does not accept Medicare, Medicaid, or private insurance. Participants between the ages of 65 and 79 were not included as most of this population qualifies for Medicare. Given that the literature suggests there is an increased risk for heart disease after the age of 60 [3], it would be prudent to include participants within the aforementioned age group in future studies. Furthermore, this particular clinic has a very large non-English-speaking population which was not included due to lack of a certified interpreter available for the purpose of this study.

The regression models are subject to several additional statistical limitations. First, VIF diagnostics revealed acceptable multicollinearity in the aggregate (VIF=1.94) and statin-user (VIF=1.25) subgroups, but the non-statin subgroup yielded a VIF of 5.16, exceeding the conventional threshold of 5.0 and reflecting the strong inter-predictor correlation between PHQ-9 and UCLA Loneliness scores in this small sample (R=0.90). Sensitivity analyses using simple linear regressions with each psychosocial predictor entered separately yielded nonsignificant associations across all lipid outcomes and subgroups, suggesting that the significance observed in the multiple regression models may reflect the joint predictive contribution of both variables rather than independent effects of either predictor alone. Second, the small subgroup sizes, particularly in the statin (n=12) and non-statin (n=14) strata, render these models susceptible to overfitting, as evidenced by negative adjusted R² values observed across several simple regression models. These models should therefore be interpreted as preliminary effect size estimates rather than stable or generalizable model fits. Third, the regression models did not adjust for potential confounders including age, sex, race, smoking status, and diabetes, all of which may independently influence lipid profiles and psychosocial measures. Notably, smoking status is a particularly salient unaddressed confounder, as current and former smokers in this sample demonstrated significantly higher PHQ-9 and UCLA Loneliness scores. The observed associations between psychosocial scores and lipid values may therefore partly reflect confounding rather than direct relationships. Future studies with larger samples should incorporate multivariate adjustment for these variables to isolate the independent contributions of depression and loneliness to cardiovascular risk factors.

Of note, the ACC ASCVD risk calculator is approved to generate a score for those between the ages of 20-79 who have had a lipid panel in the past year. However, it does not calculate a risk if TCHOL is less than 130 or if LDL is less than 70. Therefore, if a participant’s cholesterol levels did not meet these criteria, they were excluded from ASCVD risk analysis. Additionally, the calculator does not give a 10-year risk for those between the ages of 20 and 40, and does not calculate lifetime risk for those above the age of 59. Consequently, lifetime ASCVD risk was calculated only for participants who met the calculator's criteria, which significantly affected sample sizes for Pearson correlation analysis. In addition, the ASCVD risk calculator is not intended for use in those who are on statin therapy; therefore, statin users were excluded from ASCVD risk analysis as well. Future studies should consider using the new ASCVD Risk Estimator Plus (PREVENT) calculator, which incorporates renal function, BMI, and statin use in the risk estimation [25]. Multiple pairwise comparisons were also conducted without correction for multiple testing, consistent with the exploratory nature of this pilot study. Therefore, these findings should be interpreted as preliminary effect size estimates and warrant confirmation in adequately powered studies with larger, more diverse samples.

## Conclusions

No significant correlations were found between PHQ-9 scores, UCLA Loneliness scores, or ASCVD lifetime risk scores among non-statin users. This is likely attributable to the small sample size and corresponding wide confidence intervals observed across these analyses. It must be emphasized that the cross-sectional and exploratory nature of the study limited the significance of observed relationships across all analyses. In contrast, significant associations were found between psychosocial scores and lipid values: PHQ-9 and UCLA Loneliness scores were significantly associated with TCHOL in the overall sample. These associations were substantially stronger among non-statin users, where a similar pattern was also observed for HDL, although these findings may be substantially inflated due to multicollinearity. Significant associations were also found among loneliness, depression, and current/former smoking status. Taken together, these preliminary findings suggest a potential link among psychosocial factors, lipid profiles, and smoking behavior, all of which are established contributors to CVD risk, although larger studies are needed to confirm and quantify these relationships, particularly within statin-use subgroups. Future studies may consider using the PREVENT calculator to refine ASCVD risk estimation. Furthermore, future studies with larger cohorts should be performed at larger medical centers that accept public and private insurance to include a wider range of ages and socioeconomic levels. Future studies may also explore the pathophysiological mechanisms behind the impact of depression, loneliness, and lipid metabolism on heart disease, including dietary impacts, statin use as a potential moderating factor, and associations with type 2 diabetes.

## Figures and Tables

**Figure 2:**
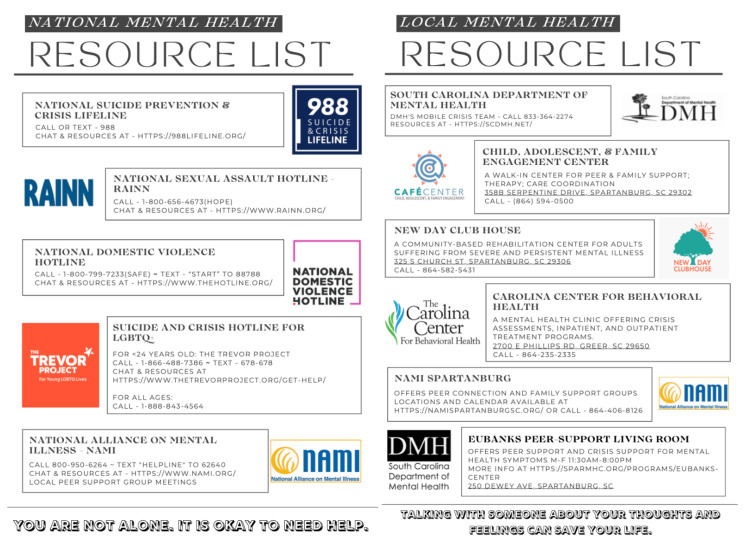
Mental Health Resource Flyer Flyer was offered to all participants. Image Credits: Hannah Elford, OMS-IV, VCOM-Carolinas (Flyer created with Canva; Canva Pty Ltd, Sydney, New South Wales, Australia). OMS: Osteopathic Medical Student, VCOM: Edward Via College of Osteopathic Medicine.

## Appendices

Appendix 1

**Table 6:** Raw Data (Psychosocial Measures and ASCVD Risk Calculations) PHQ-9: Patient Health Questionnaire-9, UCLA: University of California-Los Angeles, ASCVD: Atherosclerotic Cardiovascular Disease, N/A: not available.

Patient #	PHQ-9 Score	UCLA Loneliness Score	ASCVD 10-yr risk (%)	ASCVD Lifetime Risk (%)
1	0	47	16.1	N/A
2	3	22	14.5	69
3	0	20	0.4	27
4	20	54	16.5	N/A
5	5	22	4.6	50
6	0	20	5.1	50
7	2	27	3.4	39
8	22	72	6.9	39
9	4	26	N/A	5
10	5	36	4	69
11	6	61	14.9	N/A
12	3	24	N/A	N/A
13	8	37	4.6	39
14	2	44	7.2	69
15	9	56	34.4	N/A
16	3	33	7.7	50
17	7	28	N/A	N/A
18	9	23	N/A	N/A
19	21	53	7.1	69
20	3	25	N/A	46
21	4	31	19.6	69
22	23	67	N/A	39
23	5	27	5.1	69
24	14	46	N/A	39
25	14	56	1.5	39
26	25	37	36.7	N/A
27	4	21	10.6	39
28	18	31	2.3	39
29	1	33	3.2	50
30	9	57	N/A	50
31	4	32	12.1	N/A

Appendix 2

**Table 7:** Raw Data (Demographic Information and Various ASCVD Risk Factors) Tx: treatment; HTN: hypertension, W: White, H: Hispanic, B: Black, A: Asian, ASCVD: Atherosclerotic Cardiovascular Disease.

Age	Sex (M/F)	Race	Tx for HTN (Self-Reported)	Tx for Cholesterol (Statin Self-Reported)	Aspirin Use (Self-Reported)	Smoker	Former Smoker	Diabetes
64	F	W	FALSE	FALSE	FALSE	TRUE	FALSE	TRUE
58	M	H	TRUE	TRUE	TRUE	FALSE	FALSE	TRUE
42	F	H	FALSE	FALSE	FALSE	FALSE	FALSE	FALSE
62	F	W	TRUE	FALSE	TRUE	TRUE	FALSE	FALSE
54	F	B	TRUE	FALSE	FALSE	TRUE	FALSE	FALSE
52	F	W	TRUE	FALSE	TRUE	FALSE	TRUE	FALSE
59	F	A	FALSE	FALSE	FALSE	FALSE	FALSE	TRUE
56	F	W	FALSE	TRUE	FALSE	TRUE	FALSE	FALSE
30	M	A	FALSE	FALSE	FALSE	FALSE	FALSE	FALSE
48	M	W	TRUE	TRUE	TRUE	TRUE	FALSE	FALSE
60	M	W	FALSE	TRUE	TRUE	TRUE	FALSE	FALSE
62	M	W	TRUE	TRUE	TRUE	FALSE	TRUE	FALSE
54	F	W	FALSE	FALSE	FALSE	TRUE	FALSE	FALSE
43	M	W	TRUE	TRUE	TRUE	FALSE	FALSE	TRUE
64	F	B	TRUE	TRUE	FALSE	FALSE	TRUE	TRUE
51	F	B	TRUE	FALSE	FALSE	FALSE	FALSE	FALSE
61	M	W	TRUE	TRUE	TRUE	FALSE	TRUE	FALSE
47	M	B	TRUE	TRUE	TRUE	FALSE	TRUE	FALSE
42	M	B	FALSE	FALSE	TRUE	TRUE	FALSE	FALSE
31	M	W	TRUE	FALSE	FALSE	FALSE	FALSE	FALSE
54	M	B	TRUE	FALSE	FALSE	FALSE	FALSE	FALSE
26	F	W	FALSE	FALSE	FALSE	TRUE	FALSE	FALSE
46	M	H	TRUE	TRUE	FALSE	FALSE	FALSE	TRUE
51	F	W	TRUE	TRUE	FALSE	FALSE	FALSE	FALSE
48	F	W	FALSE	FALSE	TRUE	FALSE	TRUE	TRUE
60	M	B	TRUE	TRUE	TRUE	TRUE	FALSE	TRUE
59	F	B	FALSE	FALSE	FALSE	FALSE	TRUE	FALSE
51	F	W	FALSE	FALSE	FALSE	FALSE	FALSE	FALSE
47	M	W	FALSE	FALSE	FALSE	TRUE	FALSE	FALSE
39	F	B	FALSE	FALSE	FALSE	FALSE	TRUE	TRUE
64	F	B	TRUE	TRUE	FALSE	FALSE	TRUE	FALSE

Appendix 3

Original Survey

ASCVD QUESTIONS

Please fill out the following information to the best of your ability:

What is your current age (20-79)? ____________________

What sex were you assigned at birth (male/female/other)? __________________

What race do you identify with the most (White/African American, Other) ______________

Do you have a history of diabetes (Yes/No)? ________________

Please describe your smoking habits:

- Current

- Former

- Never

Are you being treated for high blood pressure (Yes/No)? ________________

Are you taking a statin (Yes/No)? ________________

Are you taking aspirin (Yes/No)? ________________

**Table 8:** Depression Screening (PHQ-9) PHQ-9: Patient Health Questionnaire-9.

	Not At All (0)	Several Days (1)	More Than Half the Days (2)	Nearly Every Day (3)
Little interest or pleasure in doing things				
Feeling down, depressed, or hopeless				
Trouble falling or staying asleep, or sleeping too much				
Feeling tired or having little energy				
Poor appetite or overeating				
Feeling bad about yourself – or that you are a failure or have let yourself or your family down				
Trouble concentrating on things, such as reading the newspaper or watching television				
Moving or speaking so slowly that other people could have noticed. Or the opposite – being so fidgety or restless that you have been moving around a lot more than usual				
Thoughts that you would be better off dead, or off hurting yourself				

ADDITIONAL QUESTIONS

Are you currently being treated for depression (Yes/No)? ________________

Are you currently being treated for another kind of mental health condition, other than depression (Yes/No)? ________________

What kind of mental health treatment are you receiving? (Select all that apply)

- Counseling/Therapy

- Medication/prescriptions

- None of the above

Are you interested in receiving information regarding national and local mental health resources (Yes/No)? ________________

*FOR INVESTIGATOR USE ONLY*

Date: _________________

Blood Pressure (Systolic/Diastolic): ________________

Date of Most Recent Lipid Panel: _________________

Total Cholesterol (mg/dL): ___________

HDL Cholesterol (md/dL): ___________

LDL Cholesterol (mg/dL): ___________

Appendix 4
